# Determinants of anti-S immune response at 6 months after COVID-19 vaccination in a multicentric European cohort of healthcare workers – ORCHESTRA project

**DOI:** 10.3389/fimmu.2022.986085

**Published:** 2022-09-29

**Authors:** Giulia Collatuzzo, Giovanni Visci, Francesco S. Violante, Stefano Porru, Gianluca Spiteri, Maria Grazia Lourdes Monaco, Francesca Larese Fillon, Corrado Negro, Christian Janke, Noemi Castelletti, Giuseppe De Palma, Emanuele Sansone, Dana Mates, Silvia Teodorescu, Eleonóra Fabiánová, Jana Bérešová, Luigi Vimercati, Silvio Tafuri, Mahsa Abedini, Giorgia Ditano, Shuffield S. Asafo, Paolo Boffetta, Orchestra WP5 Working Group, Carlotta Zunarelli

**Affiliations:** ^1^Department of Medical and Surgical Sciences, University of Bologna, Bologna, Italy; ^2^Section of Occupational Medicine, Department of Diagnostics and Public Health, University of Verona, Verona, Italy; ^3^Clinical Unit of Occupational Medicine, University Hospital of Verona, Verona, Italy; ^4^Unit of Occupational Medicine, University of Trieste, Trieste, Italy; ^5^Division of Infectious Diseases and Tropical Medicine, Ludwig Maximilian University (LMU) Klinikum, Munich, Germany; ^6^Department of Medical and Surgical Specialties, Radiological Sciences and Public Health, University of Brescia, Brescia, Italy; ^7^National Institute of Public Health, Bucharest, Romania; ^8^Occupational Health Department, Regional Authority of Public Health, Banská Bystrica, Slovakia; ^9^Epidemiology Department, Regional Authority of Public Health, Banská Bystrica, Slovakia; ^10^Interdisciplinary Department of Medicine, University of Bari, Bari, Italy; ^11^Stony Brook Cancer Center, Stony Brook University, Stony Brook, NY, United States

**Keywords:** vaccine, COVID – 19, serology, health care workers (HCW), immune response

## Abstract

**Background:**

The duration of immune response to COVID-19 vaccination is of major interest. Our aim was to analyze the determinants of anti-SARS-CoV-2 IgG titer at 6 months after 2-dose vaccination in an international cohort of vaccinated healthcare workers (HCWs).

**Methods:**

We analyzed data on levels of anti-SARS-CoV-2 Spike antibodies and sociodemographic and clinical characteristics of 6,327 vaccinated HCWs from 8 centers from Germany, Italy, Romania and Slovakia. Time between 1^st^ dose and serology ranged 150-210 days. Serological levels were log-transformed to account for the skewness of the distribution and normalized by dividing them by center-specific standard errors, obtaining standardized values. We fitted center-specific multivariate regression models to estimate the cohort-specific relative risks (RR) of an increase of 1 standard deviation of log antibody level and corresponding 95% confidence interval (CI), and finally combined them in random-effects meta-analyses.

**Results:**

A 6-month serological response was detected in 99.6% of HCWs. Female sex (RR 1.10, 95%CI 1.00-1.21), past infection (RR 2.26, 95%CI 1.73-2.95) and two vaccine doses (RR 1.50, 95%CI 1.22-1.84) predicted higher IgG titer, contrary to interval since last dose (RR for 10-day increase 0.94, 95%CI 0.91-0.97) and age (RR for 10-year increase 0.87, 95%CI 0.83-0.92). M-RNA-based vaccines (p<0.001) and heterologous vaccination (RR 2.46, 95%CI 1.87-3.24, one cohort) were associated with increased antibody levels.

**Conclusions:**

Female gender, young age, past infection, two vaccine doses, and m-RNA and heterologous vaccination predicted higher antibody level at 6 months. These results corroborate previous findings and offer valuable data for comparison with trends observed with longer follow-ups.

## Introduction

COVID-19 represents one of the major acute infectious threats of the XXI century. The pandemic nature of COVID-19 infection rose several challenges, leading to deep daily life changes in most populations of the world (1). The pandemic implied an urgent need for vaccines development, which first entered in use in December 2020 (2). The mRNA mechanism of newly developed vaccines, namely Comirnaty (BioNTech/Pfizer) and Spikevax (Moderna), has been largely debated. mRNA vaccines were known to be versatile and rapid to design even before COVID pandemic (3), with the benefit of a short manufacturing time matched with high efficacy, and to be overall safe (4). In many countries, health care workers (HCWs) were among the first population groups to be recommended the vaccination, given their high exposure to COVID-19 infection (5).

Once vaccines were recommended at mass level against infection spreading (6), one of the main issues became to determine their effectiveness against COVID-19 infection. Preliminary data showed that vaccines were effective against the development of symptoms and reduced the risk of infection (2, 4, 7). Indeed, immune responsiveness is necessary for a vaccine to be effective towards its target (8). The quantity of antibodies against the targeted microorganism depends on the type of vaccine and can be interpreted as an index of effectiveness of a vaccine, and the type of induced antibodies (9). In addition, subject-related factors can influence the serological response: health conditions such as immunosuppression, diabetes, autoimmune diseases and cardiovascular diseases have been described as inversely related to immune response after COVID-vaccine, while young age and female sex have been directly related to it. Baseline seropositivity was also reported as predictor of higher serological response after vaccination (10, 11). Despite several studies were conducted in occupational settings, little information is available for different job categories, where exposure to infection and subsequent development of natural antibodies may mediate the different serological level after vaccination, in particular among HCWs (12).

To date, few studies have evaluated the longitudinal immune response to COVID-19 vaccines (13–16). A recent publication reviewed the available data on duration of vaccine effectiveness, which was assessed to decrease by about 20-30% within 6 months (17).

ORCHESTRA is a multicenter prospective cohort including HCWs from multiple countries (18). This analysis within ORCHESTRA is focused on the characteristics of anti-Sars-CoV-2 Spike immune response to COVID-19 vaccines at 6 months since the 1rst dose. Previous publications based on ORCHESTA dataset reported the kinetics of antibody response to COVID-19 vaccination (19), the predictors of immunological response to vaccination (20), as well as the predictors of COVID-19 infection in HCWs by occupational factors such as use of personal protective equipment (PPE) and job title (12).

ORCHESTRA provides unique data on different job titles, COVID-19 history of the HCWs, and type of vaccine administered.

We aimed at identifying the predictors of immune response up to 6 months from vaccination, by exploring HCW-related and vaccination-related characteristics.

## Methods

ORCHESTRA comprises a prospective multicenter cohort of HCWs employed in hospitals in multiple countries (18) including over 60,000 HCWs. This analysis includes HCWs from one center in Germany (Munich), 5 centers in Italy (Bari, Bologna, Brescia, Trieste and Verona), as well as in several centers in Romania and Slovakia (the two latter treated as individual cohorts), with serological results at 6 months after first vaccination dose. Data on sociodemographic characteristics, results of PCR testing, and vaccination status, including date of vaccination doses and type, were either abstracted from medical surveillance records or collected using questionnaires or ongoing loco-regional databases. Results on level of anti-S antibodies were either collected from medical records or generated through *ad-hoc* testing. All cohorts included in the ORCHESTRA project have undergone extensive data harmonization.

The proportion of HCWs who did not develop a serological response after vaccination varied across the cohorts from 0% to 1.1%; these subjects were excluded from all analysis on serological results. The present analysis comprises 6,327 HCWs with available and positive serology results during a 6-month timeframe from 1^rst^ dose administration, defined as the interval 150-210 days (**Table 1**).

**Table 1:** Selected characteristics of the cohorts of HCW included in the analysis.

Cohort	Sources of HCW	Source of data	Time period – vaccination*
Germany-Munich	München Klinik Group, Hospital Barmherzige Brüder in München	Questionnaire and DBS data at recruitment	December 2020 – March 2021 (100%)
Italy-Bari	University Hospital of Bari	Health surveillance records	December 2020 – March 2021 (100%)
Italy-Bologna	Public hospitals and public health authority of Bologna	Health surveillance records	December 2020 – March 2021 (100%)
Italy-Brescia	Public hospitals and public health authority of Brescia	Health surveillance records	February-May 2021 (100%)
Italy-Trieste	University Hospital of Trieste	Health surveillance records	January – March 2021 (100%)
Italy-Verona	University Hospital of Verona	Health surveillance records; ongoing regional databases	December 2020 – April 2021 (100%)
Romania-Multicenter	Public health authority and institutes, medical offices, hospitals	Active recruitment	January-March 2021 (85%)
Slovakia-Multicenter	Hospitals, outpatient clinics, public health authority, social care units	Active recruitment	January – March 2021 (98%)

HCW, healthcare worker.

*The percentages refer to the number of HCWs who were vaccinated in the period indicated. The remaining HCWs were vaccinated after that period.

The primary outcome of this analysis was level of serum antibodies at six months. Methods of measurement of antibody level varied across centers and time periods; details are reported in **Supplementary Table 1**.

We conducted a two-stage analysis. In the first stage, we executed descriptive analysis of the outcome and explanatory variables. For quantitative analyses antibody levels were log-transformed to account for the skewness of the distribution. To take into account the heterogeneity in analytical methods, log-transformed results were normalized by dividing them by the center-specific standard errors. In this way, standardized serological measurements were obtained, allowing comparison across cohorts within the study population. We fitted multivariate linear regression models to estimate cohort-specific relative risks (RR) and corresponding 95% confidence intervals (CI) of an increase of one standard deviation (SD) of normalized log-transformed antibody level. Multivariate regression models, both logistic and linear, comprised sex, age, and potential determinants of levels of antibodies, including job title (technician, nurse, physician, other HCWs vs administrative personnel), time since last dose of COVID vaccine, COVID infection prior to serology (either before or after vaccination), previous positive anti-N serology (both in qualitative and quantitative terms), number of vaccine doses, type of vaccine, and BMI.

In a second phase, cohort-specific results were combined using random-effects meta-analyses (21); heterogeneity between cohort-specific results was tested using the I^2^ method (22).

Secondary analyses on vaccine type were restricted to the cohorts from Bologna and Munich.

Stata^®^ software 16 (StataCorp LP, College Station, Texas, USA) was used in the statistical analysis.

The study was approved by the Italian Medicine Agency (AIFA) and the Ethics Committee of Italian National Institute of Infectious Diseases (INMI) Lazzaro Spallanzani. Each cohort was approved by the local ethical board.

## Results

A total of 6,327 vaccinated HCWs from 8 European cohorts were included in the analysis. Selected characteristics of these HCWs are described in **Table 2**. Subjects were mostly women, with proportion ranging from 58.1% (Bari) to 81.4% (Slovakia and Romania), and older than 50 years old (from 36.8% in Bologna to 57.1% in Romania). The most frequent job titles were nurse and physician in all the cohorts, except for Slovakia where the largest category was that of other HCWs. The proportion of HCWs with a confirmed COVID-19 infection (positive by either PCR or anti-N antibodies) prior to the blood sampling was quite heterogeneous, ranging between 1.35 (Bari) to 24.1% (Brescia). Qualitative data on pre-vaccination serology were available for 5 out of 8 cohorts and showed differences among the study centers, with proportions of negative serology ranging from 51.8% (Brescia) to 90.5% (Bari). When considering type of vaccine, Comirnaty was the most commonly administered vaccine everywhere, representing 100% of the vaccinations in Bari, Verona and Trieste. Munich was the only center where a sizable proportion of subjects received other vaccines, including combinations.

**Table 2:** Selected characteristics of HCWs included in the analysis.

	Germany-Munich (%)	Italy-Bari (%)	Italy-Bologna (%)	Italy-Brescia (%)	Italy-Trieste (%)	Italy-Verona (%)	Romania-Multicenter (%)	Slovakia-Multicenter (%)
Number of HCW	292	74	2,833	253	526	2,062	210	95
Qualitative characteristics ^+^								
Sex								
Men	83 (28.42)	31 (41.9)	785 (27.7)	57 (22.5)	127 (26.8)	508 (24.6)	39 (18.6)	18 (18.9)
Women	209 (71.6)	43 (58.1)	2,048 (72.3)	196 (77.5)	347 (73.2)	1,554 (75.4)	171 (81.4)	77 (81.4)
Age group								
<= 29	64 (21.92)	13 (17.6)	421 (14.9)	47 (18.6)	31 (6.5)	285 (13.8)	10 (4.8)	11 (11.6)
30 – 39	54 (18.49)	11 (14.9)	709 (25.0)	37 (14.6)	70 (14.8)	412 (20.0)	19 (9.0)	11 (11.6)
40 – 49	48 (16.44)	15 (20.3)	659 (23.3)	59 (23.3)	126 (26.6)	448 (21.7)	61 (29.1)	24 (25.3)
>= 50	126 (43.15)	35 (47.3)	1,044 (36.8)	110 (43.5)	247 (52.1)	917 (44.5)	120 (57.1)	49 (51.6)
Job title								
Administration	NA	3 (4.4)	103 (3.6)	32 (12.6)	19 (4.0)	211 (10.2)	23 (10.9)	17 (17.9)
Physician	NA	33 (48.5)	621 (22.0)	49 (19.4)	54 (11.3)	480 (23.3)	93 (44.3)	11 (11.6)
Nurse	NA	19 (27.9)	1,119 (39.6)	84 (33.2)	203 (42.6)	806 (39.1)	29 (13.8)	20 (21.0)
Technician	NA	\	273 (9.7)	20 (7.9)	26 (5.5)	233 (11.3)	58 (27.6)	13 (13.7)
Other HCW	NA	13 (19.1)	709 (25.1)	68 (26.9)	174 (36.6)	332 (16.1)	7 (3.3)	34 (35.8)
Previous Covid-19 infection **								
No	262 (90.97)	73 (98.6)	2,609 (92.1)	192 (75.9)	432 (82.1)	1,752 (85.0)	182 (86.7)	81 (85.3)
Yes	26 (9.03)	1 (1.3)	224 (7.9)	61 (24.1)	94 (17.9)	310 (15.0)	28 (13.3)	14 (14.7)
Qualitative pre-vaccination serology								
Negative	NA	67 (90.5)	1,045 (88.1)	29 (51.8)	142 (69.3)	1,526 (89.5)	NA	NA
Positive	NA	7 (9.5)	141 (11.9)	27 (48.2)	63 (30.7)	179 (10.5)	NA	NA
Type of vaccine								
Comirnaty	198 (70.4)	74 (100.0)	2,732 (97.3)	247 (97.6)	526 (100.0)	2,062 (100.0)	206 (98.1)	94 (98.9)
Spikevax	24 (8.54)	0 (0.0)	74 (2.6)	1 (0.4)	0 (0.0)	0 (0.0)	2 (0.9)	0 (0.0)
Vaxzevria	11 (3.91)	0 (0.0)	3 (0.1)	5 (2.0)	0 (0.0)	0 (0.0)	2 (0.9)	1 (1.0)
Vaxzevria + Comirnaty	45 (16.01)	0 (0.0)	0 (0.0)	0 (0.0)	0 (0.0)	0 (0.0)	0 (0.0)	0 (0.0)
Vaxzevria + Spikevax	3 (1.07)	0 (0.0)	0 (0.0)	0 (0.0)	0 (0.0)	0 (0.0)	0 (0.0)	0 (0.0)
Number of doses								
1 dose received	11 (3.77)	0 (0.0)	18 (0.6)	44 (17.4)	34 (6.5)	92 (4.5)	0 (0.0)	0 (0.0)
2 doses received	281 (96.23)	74 (100.0)	2,815 (99.4)	209 (82.6)	492 (93.5)	1,970 (95.5)	210 (100.0)	95 (100.0)
Quantitative characteristics *								
Standardized quantitative serology at 6-month								
Mean (SD)	3.72 (0.06)	8.40 (0.12)	7.31 (0.02)	6.02 (0.06)	6.29 (1.00)	6.51 (1.00)	6.32 (0.99)	8.51 (0.10)
Quantitative pre-vaccination serology								
Mean (SD)	NA	92.03 (605.79)	\	44.02 (66.85)	29.88 (75.89)	0.86 (3.69)	NA	NA
Standardized quantitative pre-vaccination serology†								
Mean (SD)	NA	0.55 (1.00)	\	0.30 (1.38)	4.23 (1.00)	-1.11 (1.00)	NA	NA
**Days between pre-vaccination serology and serology at 6-month**								
Mean (SD)	NA	204.19 (6.90)	360.56 (100.71)	299.75 (27.37)	422.42 (128.75)	294.69 (48.14)	NA	NA
Range	NA	(186, 254)	(155, 626)	(200, 329)	(157, 628)	(182, 494)	NA	NA
**Days between 1^st^ dose and serology at 6-month**								
Mean (SD)	178.52 (16.81)	203.31 (2.10)	176.94 (17.67)	184.95 (15.11)	172.95 (19.00)	199.41 (13.22)	196.68 (13.12)	160.98 (15.60)
Range	(150, 210)	(194, 208)	(150, 210)	(150, 210)	(150, 210)	(151, 210)	(154, 210)	(150, 209)

+, Frequency and percentage for the categorical variables are reported.

*, Mean and SD for the continuous variables are reported.

NA, no data available.

** In Germany-Munich cohort, previous Covid-19 infection has been detected by at least one positive PCR or Anti-N.

†Anti-N antibodies. The same standardization method was applied as for the 6-month serology. In other center, pre-vaccination status was assessed by using PCR.

numbers may not sum to the total because of missing values.

Overall, 199 HCWs (3.2%) received only one dose of vaccine. This proportion was largest in Brescia (17.4%). Instead, 100% of the subjects from Bari, Slovakia and Romania cohorts completed the two-dose vaccination course. Pre-vaccination anti-N antibody level was provided by 4 cohorts and the intervals varied by cohort (**Table 2****)**. The mean timeframe between first vaccine dose and blood sample varied between 161.0 (Slovakia) to 203.3 days (Bari), and the overall mean was 185.1, within the predefined range of 150-210 days.

**Supplementary Table 2** illustrates the serology level distribution categorized by sex, age and cohort. The results of the meta-analysis for the determinants of serology response at 6 months are reported in **Table 3**, and the corresponding cohort-specific results are reported in **Supplementary Figures 1–7**. Overall, women were more likely to develop a higher antibody level than men (RR of an increase of one SD of normalized log-transformed antibody level 1.10, 95% CI 1.00-1.21, p-heterogeneity 0.1). Cohort-specific results are shown in **Supplementary Figure 1**, the RRs ranged from 0.91 to 1.51. Ageing was inversely related to serologic response in all the cohorts, with RR=0.87 for a 10-year increase in age (95% CI 0.83-0.92, p-heterogeneity 0.003). Cohort-specific results are shown in **Supplementary Figure 2**; RRs were all below 1 and ranged from 0.76 to 0.98. Job title (seven cohorts) was not associated to the serology level, either in the meta-analysis (**Table 3**) or in cohort-specific analyses (details not shown).

**Table 3:** Determinants of standardized antibody level at 6-month.

	RR	95% CI	p-value
Gender* [all]			
Men	1 (Ref)		
Women	1.10	1.00- 1.21	0.041
Age* [all]			
10 years increase	0.87	0.83-0.92	<0.001
Job title* [It-Ba, It-Bo, It-Br, It-Ts, It-Vr, Ro-Mc, Sk-Mc]			
Administration	1 (Ref)		
Physician	1.00	0.89-1.13	0.990
Nurse	0.93	0.81-1.07	0.302
Technician	1.04	0.92-1.18	0.495
Other HCW	1.03	0.91-1.16	0.690
Previous Covid-19 infection*¥ [all]			
No	1 (Ref)		
Yes	2.26	1.73-2.95	<0.001
Number of doses* [Ge-Mu, It-Bo, It-Br, It-Ts, It-Vr]			
1 dose received	1 (Ref)		
2 doses received	1.50	1.22-1.84	<0.001
Days between last dose and serology at 6-month* [It-Ba, It-Bo, It-Br, It-Ts, It-Vr, Ro-Mc, Sk-Mc]			
10 days increase	0.94	0.91-0.97	<0.001
Type of vaccine* [Ge-Mu, It-Bo, It-Br, Ro-Mc]			
mRNA	1 (Ref)		
Viral vector	0.58	0.27-1.23	0.154
Qualitative pre-vaccination serology*¢ [It-Ba, It-Bo, It-Br, It-Ts, It-Vr]			
Negative	1 (Ref)		
Positive	1.85	1.35-2.52	<0.001
Standardized quantitative pre-vaccination serology*¢ [It-Ba, It-Br, It-Ts, It-Vr]			
**1 SD increase in ln(AB)**	1.19	1.05-1.35	0.006
**Days between pre- vaccination serology and serology at 6-month† [It-Ba, It-Br, It-Ts, It-Vr]**			
30 days increase	1.09	0.97-1.23	0.129
BMI* [Ro-Mc, Sk-Mc]			
1 unit increase	1.02	0.99-1.04	0.186

Ge-Mu, Germany-Munich; It-Ba, Italy-Bari; It-Bo, Italy-Bologna; It-Br, Italy-Brescia; It-Ts, Italy-Trieste; It-Vr, Italy-Verona; Ro-Mc, Romania-Multicenter; Sk-Mc, Slovakia-Multicenter; RR, relative risk; CI, confidence interval; BMI, body mass index; Ref, reference category.

*Adjusted by age, gender, job title previous Covid-19 infection, number of doses and days between last dose and serology at 6-month (excluding variable itself).

¥ In Germany-Munich cohort, previous Covid-19 infection has been detected by at least one positive PCR or Anti-N. In other center, pre-vaccination status was assessed by using PCR.

†Adjusted by age, gender, job title, previous Covid-19 infection, number of doses, and standardized quantitative pre-vaccination serology.

¢Based on anti-N antibodies.

We found a RR of 2.26 (95% CI 1.73-2.95, p-heterogeneity < 0.001, all eight cohorts) for previous COVID-19 infection; cohort-specific RRs ranged from 1.01 to 4.95, with one outlying result from Bari, which however was based on a single HCW with previous COVID-19 infection (**Supplementary Figure 3**). A RR of 1.50 (95% CI 1.22-1.84, five cohorts) was detected for two vs one dose of vaccine. A 10-day increase since last dose (seven cohorts) showed significant probability of lower level of antibodies (RR 0.94, 95% CI=0.91-0.97).

Viral-vector vaccines (four cohorts) resulted in a non-significant lower probability of increased serological response (RR 0.58; 95% CI=0.27-1.23). HCWs who had a positive or higher serology level before vaccination had significantly a higher probability on an increased level respectively (RR=1.85, 95% CI=1.35-2.52) and (RR=1.19, 95% CI=1.05-1.35). (**Supplementary Figures 6, 7**). No difference was found based on 30 days increase in the interval since pre-vaccination serology (four cohorts): results were quite inconsistent between the cohorts. When the analysis was stratified by both infection and vaccination status, HCWs reporting history of COVID infection and administered with two doses had higher antibodies than those with no infection and one only dose (RR=23.41, 95% CI=0.46-1194.51, based on 5 cohorts). No relation was found with increasing BMI based on Slovakia and Romania cohorts.

We performed separate analyses within single centers with available data on different vaccine types, namely Italy-Bologna and Germany-Munich (**Table 4**). When comparing the different vaccines in the Italy-Bologna cohort, a higher immunogenicity was found for Spikevax against Comirnaty, up to a RR of 2.05 (p<0.001). Vaxzevria (n=74) resulted to be associated to higher level of antibodies too, but without significance (RR=1.62, p=0.31); this latter result is hampered by the very small number of HCWs receiving this vaccine (n=3). The analysis of the Germany-Munich cohort provided slightly different results from that of Bologna: Spikevax was not significantly associate to a quantitative immune response (RR=1.23, p=0.13); Vaxzevria was less able to induce serological response (RR=0.50, p=0.019). Compared to homologous Comirnaty vaccination, heterologous vaccination with Vaxzevria & Comirnaty and that with Vaxzevria & Spikevax were significantly more likely to produce higher immune response, with RR of 2.35 (p<0.001) and 2.05 (p=0.017) respectively, with an overall RR for heterologous vs. homologous vaccination equal to 2.46 (95% CI 1.87-3.24).

**Table 4:** Relative risk for type of vaccine.

Type of vaccine	RR		95% CI		p-value	
Germany, Munich	
Comirnaty	1 (Ref)		–		
Spikevax	1.23		[0.85, 1.77]		0.1	
Vaxzevria	0.50		[0.28, 0.89]		0.02	
Vaxzevria + Comirnaty	2.35		[1.75, 3.14]		<0.001	
Vaxzevria + Spikevax	2.05		[1.14, 3.71]		0.02	
Italy, Bologna						
Comirnaty	1 (Ref)		–			
Spikevax	2.05		[1.61, 2.60]		<0.001	
Vaxzevria	1.62		[0.63, 4.16]		0.3	

RR, relative risk adjusted by sex, age, job title, time since 1^st^ dose, previous Covid-19 infection and number of doses; CI, confidence interval; Ref, reference category.

## Discussion

The assessment of effectiveness of vaccines against covid-19 infection has represented an important research objective since their development. This paper describes the determinants of qualitative and quantitative immune response to COVID-19 vaccines at 6 months from the 1^st^ dose.

We analyzed consecutive serologies of more than 6300 HCWs from 8 cohorts being vaccinated with at least 1 dose and with available information on immunization status at 6 months.

The analyses showed that women had higher serological responses at 6 months, while ageing was inversely related to it. These data agree with several other studies (23–29) and are in line with the sex-related dimorphism of immune response (30).

As expected, we observed a reduction in the serological response at 6 months, as well as negative trend when analyzing HCWs who were tested for antibodies at increasing time intervals since the last dose of vaccine, suggesting the progressive waning of serology level. This is consistent with previous findings (23). According to Li et al. (23), the intensities of CD4+ T cell responses to inactivated vaccine antigens were lower after 30 days from the 1^st^ dose compared to subjects inoculated less than 30 days before blood sampling.

A 15-times decline of the serological level at 5-months after Comirnaty vaccine was observed among 100 HCWs (31), while the duration of Spikevax was stated to be at least 209 days based on analyses of 33 healthy adults’ serum (32). Another study on 151 HCWs measured antibodies level after 1^rst^ dose, and 1 and 3 months after 2^nd^ dose of mRNA vaccines; the results were a lower response among older subjects and the decline in the serological levels at 3 months in all the participants. Similar results were reported in studies from Portugal (33) and Italy (34).

With regard to past COVID-19 infection, our study showed that HCWs with COVID-19 infection prior to vaccination were more likely to maintain positive responses at 6-months, also corresponding to higher immunogenicity levels. Antibodies development in absence of vaccination indicates a past contact with the correspondent pathogen. Indeed, several studies (35–37) described a higher immune response to COVID-19 vaccines in subjects with history of infection, also with a faster serological rise (36). In specific, previously infected HCWs may develop higher neutralizing antibody titers than those with a negative history of infection (38).

The quantity of neutralizing antibodies is highly correlated with the protective effect of vaccination and its durability (39). Antibody titers do not correspond to neutralizing titers, which may be relatively high and effective despite low absolute level of antibodies. The qualitative analyses partially overcome this limit, but further studies measuring the level of neutralizing antibodies and calculating their proportion among the total antibodies, as well as their relation with possible outcomes (e.g., infection, symptoms, hospitalization, reinfection) are needed.

Different immune responses were described in not infected (naïve) compared to pre-immune subjects by Forgacs et al. (35), who assessed the post-vaccination neutralization titers for immunologically naïve subjects to range from 1:5–1:400, against 1:400–1:3200 in pre-immune individuals. The serological protection conferred by vaccination was significantly more robust compared to antibodies induced by natural viral infection, as confirmed by a more recent publication (40). Moreover, vaccination elicited higher antibody titers in participants who were pre-immune to SARS-CoV-2 compared to naïve ones. The robust and immediate recall of high affinity antibodies may be attributed to memory B cell mediated processes. This highlights the importance of vaccination and booster administration, given the short-term protection and the frequent reinfections.

Zhong et al. (16) established a cohort of 3500 HCWs to compare antibody durability induced by mRNA vaccines in individuals with or without history of infection. A negative trend was found for the serological levels among participants who had never been infected, with the adjusted median antibody measurements scaling from 8.7 at one month, to 7.3 at three months, and down to 4.6 at six months after vaccination (16). Conversely, those with prior infection maintained higher postvaccination adjusted median antibody measurements by an absolute difference of 1.25 at one month, 1.42 at 3 months, and 2.56 at six months (16). Moreover, individuals who tested positive for infection more than 90 days before vaccination had higher postvaccination adjusted antibody measurements compared with those infected up to 90 days before vaccination, demonstrating also that a longer interval between infection and first vaccine dose may enhance the antibody response (16). Tanunliong and coauthors (37) have found that serological levels had still not significantly decreased after seven months from infection, while Edridge et al. (41) described a short-lasting natural immunogenicity, based on the common occurrence of coronaviruses reinfection within 12 months observed in a prospective study following up 10 healthy individuals over 35 years through blood collection every 3 months before 1989 and every 6 months afterwards. The effectiveness of Comirnaty was studied on about 3,4000,000 subjects, with a declining in the immune coverage varying from 88% after 1 months to 43% after 5 months from the second dose. Also, the authors hypothesize the decline of effectiveness is due to waning immunity rather than delta variant, as the protection against this latter considering hospital admission resulted to last around 6 months (15). Besides qualitative studies, few quantitative studies have been published, such as the one by Suthar et al. (14), which described the substantial waning of antibodies after six months from the double dose of Comirnaty. These results overall agree with our findings. Also, they corroborate the hypothesis of the third dose as useful to maintain antibody levels above the effectiveness threshold (13).

With regard to job title, no difference was found in our analysis. This is not unexpected, as far as we accounted for major confounders including previous COVID-19 infection. In fact, physician and nurses were more prone to get the infection because of higher exposure to patients compared to administrative workers or technicians. While to our knowledge this is the first study to report different levels of antibodies at 6 months from COVID-19 vaccine by job title, a previous analysis from ORCHESTRA’s Italian cohorts showed no difference in the risk of infection by occupational category, nor among HCWs working in a COVID-19 designated department (12).

Overall, our analysis showed mRNA vaccines confer higher protection than viral vector vaccine. The analysis within the Bologna Munich cohort showed some variability in the vaccines’ effectiveness, with Spikevax apparently stronger than Comirnaty. Indeed, literature reports that the higher effectiveness of mRNA vaccines may be related to the capacity to induce persistent germinal center B cell response (36). Moreover, a higher ability to stimulate an immune response was found in relation to heterologous vaccination (Vaxzevria + Comirnaty/Spikevax), with an overall 2.4-fold higher likelihood of positive immunization compared to homologous vaccination, consistently with previous findings (42).

A recent review compared the different available vaccines for COVID-19 to 2021, reporting Comirnaty and Spikevax having the higher efficacy (29). Authors highlighted the importance of the sequential immunization strategy, following the example of a potential HIV vaccination scheme: the heterogeneous prime boosts confer substantial protection and can increase the intensity and breadth of the immune response, despite no single HIV vaccine provides effective protection. Indeed, the stimulation of the immune response through different types of vaccines against the same pathogen seems to enhance the likelihood of coverage for highly mutant viruses (such as HIV, influenza and coronavirus) by providing broader protection against variants (43). Antibodies able to protect against multiple strains of a mutable pathogen are called “broadly neutralizing antibodies”, whose production is a major aim of vaccination strategy.

Several studies agree around the beneficial effects of changing prime-boost immunization strategies by altering mutational distances, concentrations and other features of the immunogens.

Also, different trends in immunogenicity have been described for the different types of vaccine. For example, mRNA vaccines showed to induce a rapid and high immune response, but with a consistent decline in the following 6-8 months, while Janssen corresponded to lower but relatively stable antibodies (13).

The results we presented are based on serologies obtained through different methods of detection. This limitation was addressed by using a statistical approach allowing to standardized serological levels, making the results by specific cohorts comparable. Indeed, all the differences found in the present analysis must be interpreted as representing real phenomena rather than being attributed to differences in serological testing across the cohorts. Despite one would ideally test all study participants with the same methods under the same conditions, this is unfeasible in a large international cohort such as the one we have studied. Given the global connotation of COVID-19 infection, this approach allows to overcome potential heterogeneity in data collection methods and to make data comparable from all over the world.

We could establish several associations with 6 months immunization, despite the information were sometimes missing for some cohorts. Data on COVID-19 infection used in this analysis were based on PCR testing for all HCW, except a small proportion of subjects on the Munich cohort for whom it was based on anti-N serology. In a sensitivity analysis excluding the Munich cohort, the results for pre-vaccination COVID-19 infection did not change compared to the main analysis. The cohort from Brescia was the only one with a substantial number of HCW tested both with PCR and anti-N antibodies. The two methods obtained similar results on the level of post-vaccination anti-S serology.

Also, adjustments were made to account for the reciprocal confounding of the determinants considered in the analyses, including job title. In fact, serological pattern by job title was also described. When interpreting the results on vaccines immunogenicity, geographical and ethnical aspects should be taken into account (44): the vaccines efficacy has been reported to be impaired by locally circulating variants; also, different level of efficacy of Comirnaty have been described by ethnicity. This limits the generalizability of our results, even if analyses were adjusted by study center. Moreover, this study may not be fully comparable to those based on the general-population, since HCWs are a population at higher risk of exposure, were among the first to be vaccinated, and HCWs can be subject to behavioral and lifestyle confounders which we did not accounted for. Also, we did not consider clinical conditions of the participants, which may have played a role. Another limitation is that date of previous COVID-19 infection was not available on subjects with pre-vaccination N-Ig results, preventing us from estimating the decline in antibody levels from a possible previous infection. Lastly, the results by type of vaccine are impaired by small numbers of subjects with available results, limiting the possible comparisons, especially when considering viral vector vaccines.

This study represents an important source of information to understand the effectiveness and the duration of immune response acquired by vaccination. Our data provide important support to previous findings on the ability of different types of vaccines to elicit immunogenicity, and show on a large multicentric study the time-trends of their immunological effect over six months. This analysis adds further useful information to help in the prioritization of candidates for vaccination campaign.

Next steps from the ORCHESTRA project will be to investigate the lifestyle and sociodemographic determinants of immune response and duration, as well as proceeding the analysis on temporal trends of serological levels.

## Orchestra WP5 Working Group

Carlotta Zunarelli (1), Roberta Bonfiglioli (1), Angela Carta (2), Giuseppe Verlato (12), Giuseppe Lippi (13), Davide Gibellini (14), Maria Diletta Pezzani (15), Lorena Torroni (12), Michael Hoelscher (5), Andreas Wieser (5), Christina Reinkemeyer (5), Michael Plank (5), Ivan Noreña (5), Raquel Rubio-Acero (5), Simon Winter (5), Mihaela Leustean (7), Ovidiu Perseca (7), Madalina Ipate (7), Agripina, Rascu (16), Jozef Strhársky (17), Petra Hellebrandt (18), Daniela Križanová (19), Marianna Mrázová (20), Luigi De Maria (10), Stefania Sponselli (10), Pasquale Stefanizzi (10), Antonio Caputi (10). ^12^Unit of Epidemiology and Medical Statistics, Department of Diagnostics and Public Health, University of Verona, Verona, Italy, ^13^Section of Clinical Biochemistry, University of Verona, Verona, Italy, ^14^Section of Microbiology, University of Verona, Verona, Italy, ^15^Infectious Disease Unit, University Hospital of Verona, Verona, Italy, ^16^Department of Internal Medicine, University of Medicine and Pharmacy Carol Davila, Bucharest, Romania, ^17^Medical Microbiology Department, Regional Authority of Public Health, Banska´ Bystrica, Slovakia, ^18^Health Promotion Department, Regional Authority of Public Health, Banska´ Bystrica, Slovakia, ^19^Hygiene Department, National Institute for Cardiovascular Diseases, Bratislava, Slovakia, ^20^Public Health Institute, St. Elizabeth University of Health and Social Work, Bratislava, Slovakia.

## Data availability statement

The raw data supporting the conclusions of this article will be made available by the author upon reasonable request.

## Ethics statement

The studies involving human participants were reviewed and approved by Italian Medicine Agency (AIFA), Ethics Committee of Italian National Institute of Infectious Diseases (INMI) Lazzaro Spallanzani. The patients/participants provided their written informed consent to participate in this study.

## Author contributions

GC, GV, and PB conceived and designed the study. PB coordinated the international collaboration. GV, FV, and PB coordinated the study in Bologna, Italy. SP, GS, and MM coordinated the study in Verona, Italy. FF and CN coordinated the study in Trieste, Italy. CJ and NC coordinated the study in Munich, Germany. GP and ES coordinated the study in Brescia, Italy. DM and STe coordinated the study in Romania. EF and JB coordinated the study in Slovakia. LV and STa coordinated the study in Bari, Italy. MA, GD, and SA performed the statistical analysis. GC and PB supervised the statistical analysis. GC and PB drafted the manuscript, with substantial input from NC. All authors contributed to the article and approved the submitted version.

## Funding

This project has received funding from the EU Horizon 2020 research and innovation programme under the ORCHESTRA project Grant Agreement No. 101016167. The cohort from Verona is funded by the Regional Health Authority (Azienda Zero), Veneto Region, Italy.

## Acknowledgments

Bologna, Italy cohort: Laboratory of Microbiology of the University Hospital. Trieste, Italy cohort: All personnel of the Clinical Unit of Occupational Health, Laboratory of Virology and Laboratory of Microbiology of the University Hospital. Verona, Italy cohort: General Management, Medical Management, and all personnel of the Units of Occupational Health, Laboratory Medicine and Microbiology and of University Hospital of Verona and all personnel of the Unit of Epidemiology and Medical Statistics, University of Verona. Slovakia cohort: Alena Koščálová, Infectology Clinic, University Hospital, Bratislava; Oto Osina, Occupational Medicine Clinic, University Hospital, Martin; Zuzana Sirotná, Laboratory Dpt, Public Health Authority of the Slovak Republic, Bratislava, Jarmila Beláková and co-workers, Occupational health Dpt., Regional Authority of Public Health, Banská Bystrica; Alexandra Bražinová, Institute of Epidemiology, Faculty of Medicine Comenius University, Bratislava.

## Conflict of interest

The authors declare that the research was conducted in the absence of any commercial or financial relationships that could be construed as a potential conflict of interest.

## Publisher’s note

All claims expressed in this article are solely those of the authors and do not necessarily represent those of their affiliated organizations, or those of the publisher, the editors and the reviewers. Any product that may be evaluated in this article, or claim that may be made by its manufacturer, is not guaranteed or endorsed by the publisher.
