# Supplementary material for: Determinants of anti-S immune response at 6 months after COVID-19 vaccination in a multicentric European cohort of healthcare workers – ORCHESTRA project
Source: Front Immunol. 2022 Sep 29;13:986085. doi: 10.3389/fimmu.2022.986085 (PMC9559243; doi:10.3389/fimmu.2022.986085)
Supplement: Supplementary file 1 [file DataSheet_1.pdf]

Supplementary Table 1. Analytical methods used to measure SARS-CoV-2 antibody level.

Cohort	Type of serology test	**Unit of measurement**	Mean (SD) of serological level*	Mean (SD) of standardized serological level*†
Germany-Munich	Roche Elecsys ® anti SARS CoV2 S -	U/mL	47.35 (2.68)	3.72 (0.06)
Italy-Bari	Abbot SARS-COV-2 IgG II Quant test	AU/ml	1711.04 (252.94)	8.45 (0.13)
Italy-Bologna	Ab anti SARS CoV-2 S (RBD) IgG ECLIA	BAU/ml	1062.70 (17.96)	7.38 (0.02)
Italy-Brescia	Roche Elecsys® anti SARS CoV2 S	U/mL	2349.10 (162.77)	6.07 (0.06)
Italy-Trieste	CMIA anti S1-RBD	RLU	3383.6 (471.98)	6.15 (0.06)
Italy-Verona	CLIA trimeric S IgG	BAU/ml	868.39 (39.53)	6.39 (0.02)
Romania-Multicenter	Abbot SARS-COV-2 IgG II Quant test	AU/mL	1702.06 (3674.69)	6.35 (0.99)
Slovakia-Multicenter	QuantiVac ELISA (IgG) EUROIMMUN	RU/mL	904.82 (95.23)	8.58 (0.19)

* Adjusted by age, according to the Standard European Population
† Standardized according to the formula: ln(AB) / sd[ln(AB)], where AB stands for antibody level.
U=unitss
AU=antibody units
BAU=binding antibody units
RLU=relative light units
RU=relative units

Supplementary Table 2. Standardized serology level distribution by cohort, sex and age

Variable	Standardized. quantitative serology level – Mean (SD)							
Cohorts	Germany-Munich	Italy-Bari	Italy-Bologna	Italy-Brescia	Italy-Trieste	Italy-Verona	Romania-Multicenter	Slovakia-Multicenter
Sex*	-	-	-	-	-	-	-	-
Male	3.60 (0.10)	8.37 (0.19)	7.29 (0.04)	6.12 (0.13)	6.10 (0.12)	6.35 (0.04)	6.07 (0.81)	8.42 (0.46)
Female	3.83 (0.07)	8.52 (0.17)	7.41 (0.02)	6.06 (0.07)	6.15 (0.08)	6.40 (0.03)	6.41 (1.03)	8.59 (0.21)
Age (yrs)	-		-	-	-	-	-	-
≤ 29	4.05 (0.09)	8.77 (0.30)	7.74 (0.04)	6.30 (0.12)	6.52 (0.17)	6.62 (0.04)	6.46 (0.54)	8.70 (0.33)
30-39	3.70 (0.18)	8.42 (0.15)	7.48 (0.03)	5.82 (0.17)	6.17 (0.12)	6.41 (0.04)	6.16 (0.95)	8.41 (0.20)
40-49	3.51 (0.13)	8.81 (0.23)	7.17 (0.03)	5.89 (0.13)	6.00 (0.08)	6.22 (0.04)	6.32 (1.04)	8.45 (0.18)
≥ 50	3.62 (0.09)	8.08 (0.18)	7.12 (0.03)	6.04 (0.10)	5.86 (0.07)	6.25 (0.03)	6.33 (1.02)	8.52 (0.16)

* Adjusted by age, according to the Standard European Population.

Supplementary Figure 1. Relative risks and 95% confidence intervals of one SD increased in normalized antibody level by sex; results of individual cohorts and meta-analysis

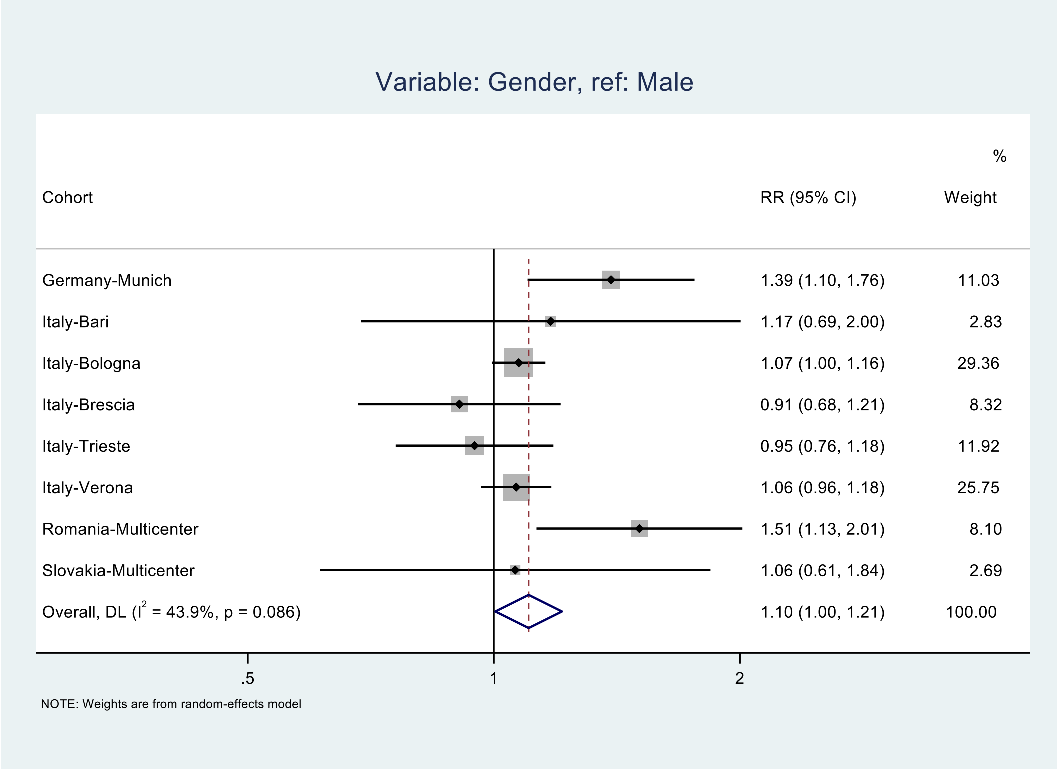

Supplementary Figure 2. Relative risks and 95% confidence intervals of one SD increased in normalized antibody level, by age; results of individual cohorts and meta-analysis.

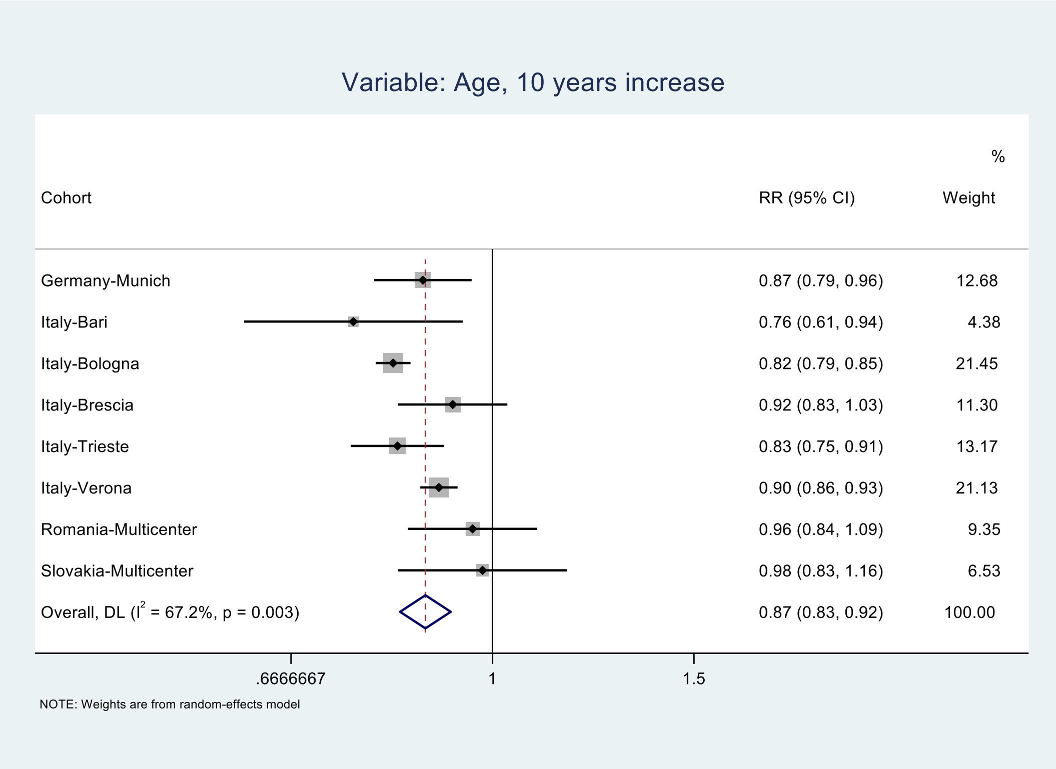

Supplementary Figure 3. Relative risks and 95% confidence intervals of one SD increased in normalized antibody level by previous COVID infection; results of individual cohorts and meta-analysis

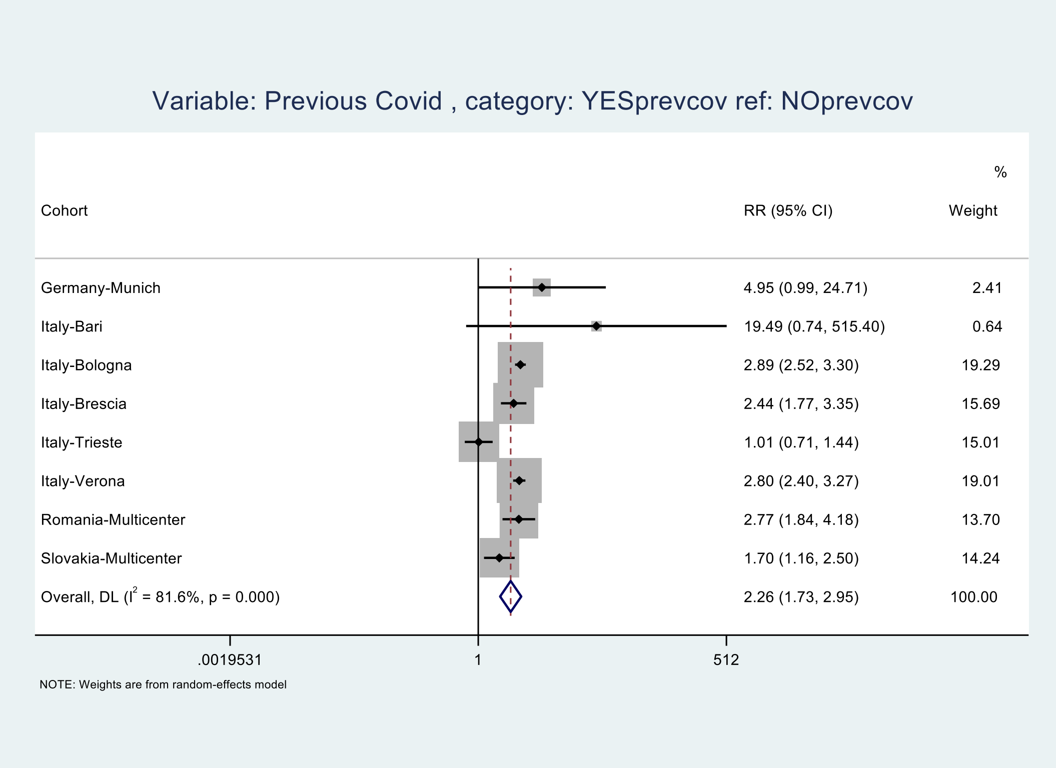

Supplementary Figure 4. RR for number of vaccine doses; results of individual cohorts and meta-analysis

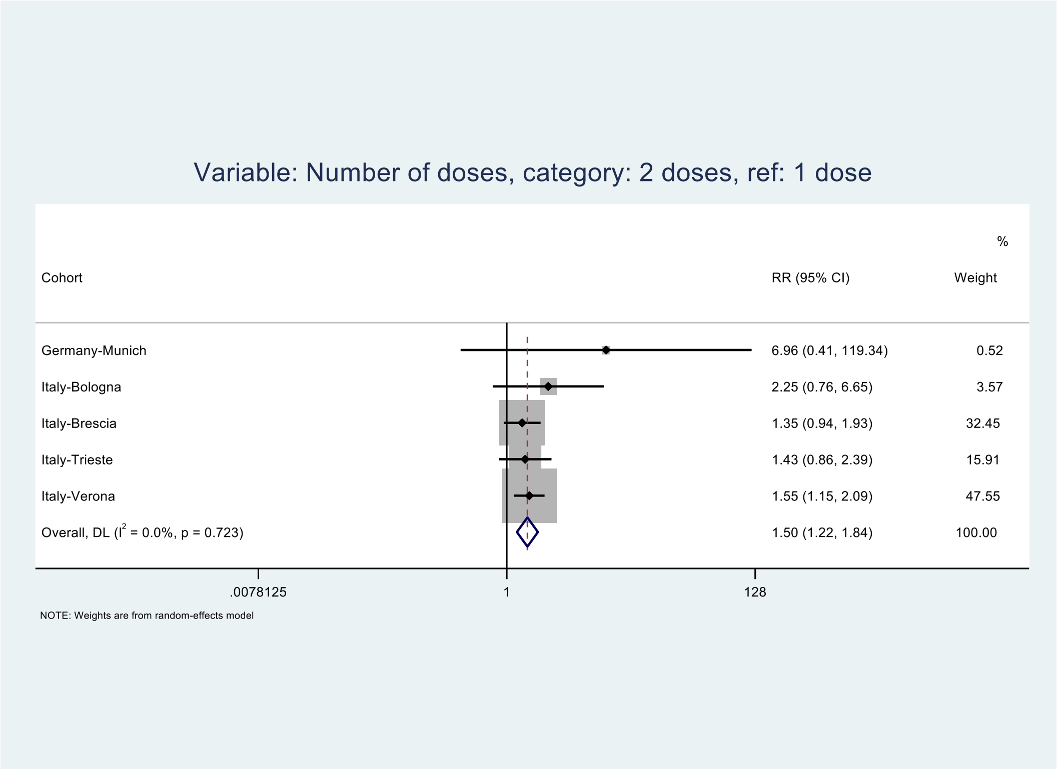

Relative risks and 95% confidence intervals of one SD increased in normalized antibody level

Supplementary Figure 5. RR for type of vaccine; results of individual cohorts and meta-analysis

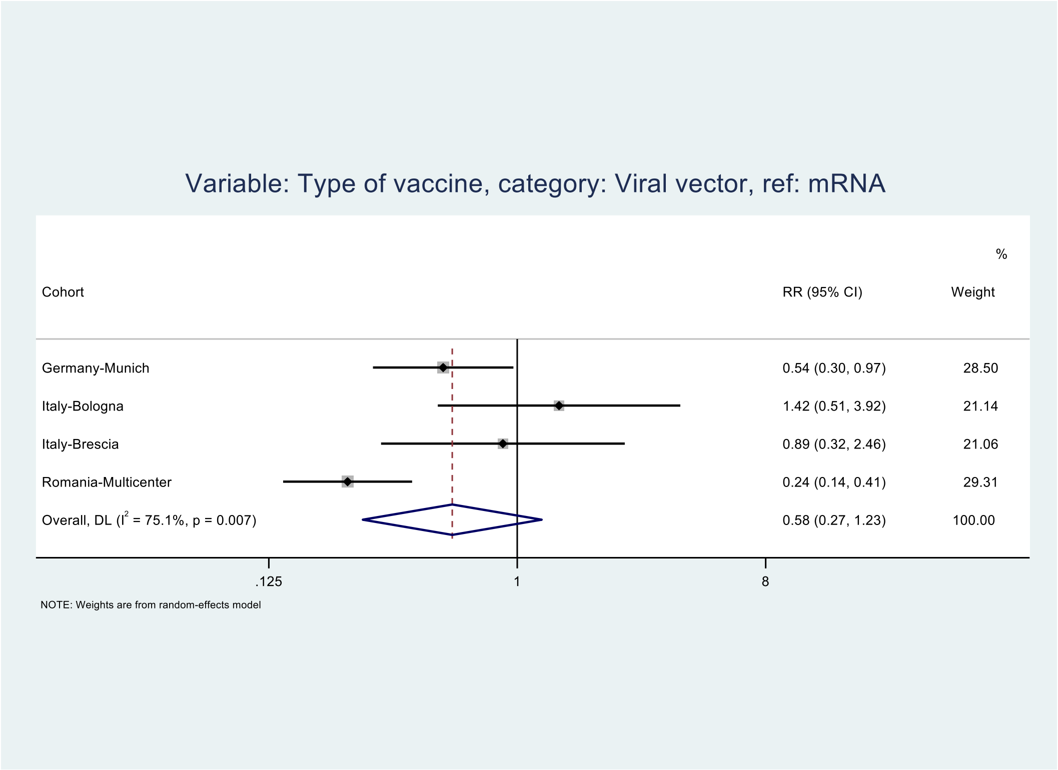

Relative risks and 95% confidence intervals of one SD increased in normalized antibody level

Supplementary Figure 6. RR for pre-vaccination serology (qualitative); results of individual cohorts and meta-analysis

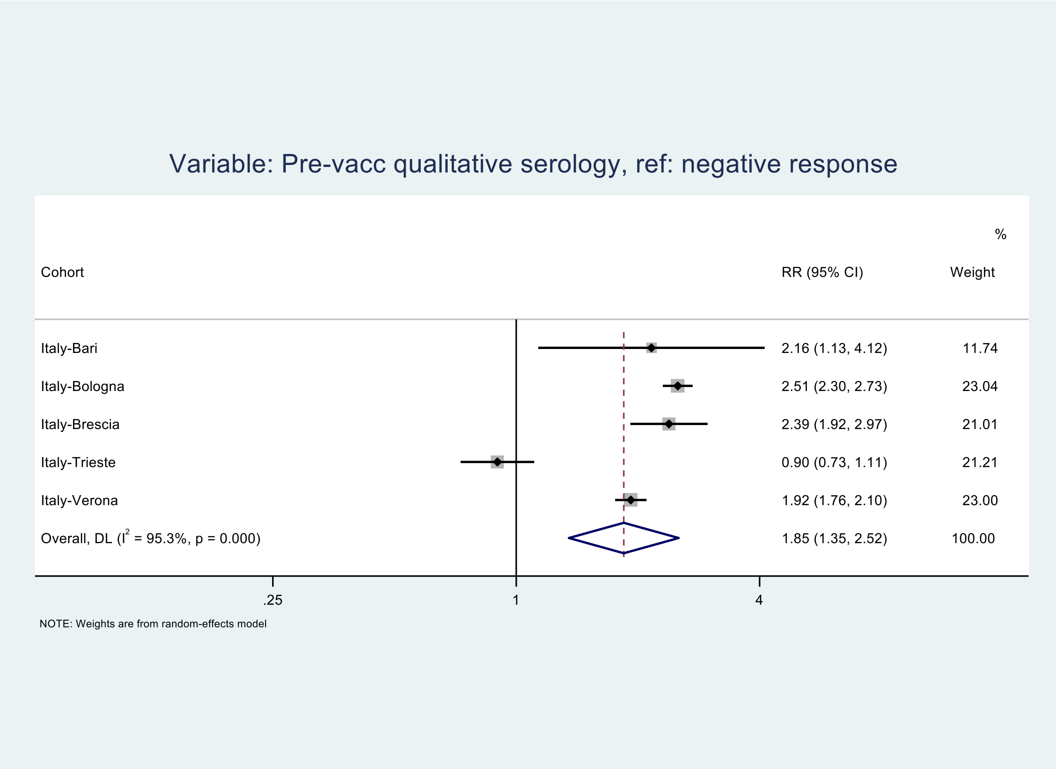

Relative risks and 95% confidence intervals of one SD increased in normalized antibody level

Supplementary Figure 7. RR for pre-vaccination serology (one SD increase in normalized antibody level); results of individual cohorts and meta-analysis

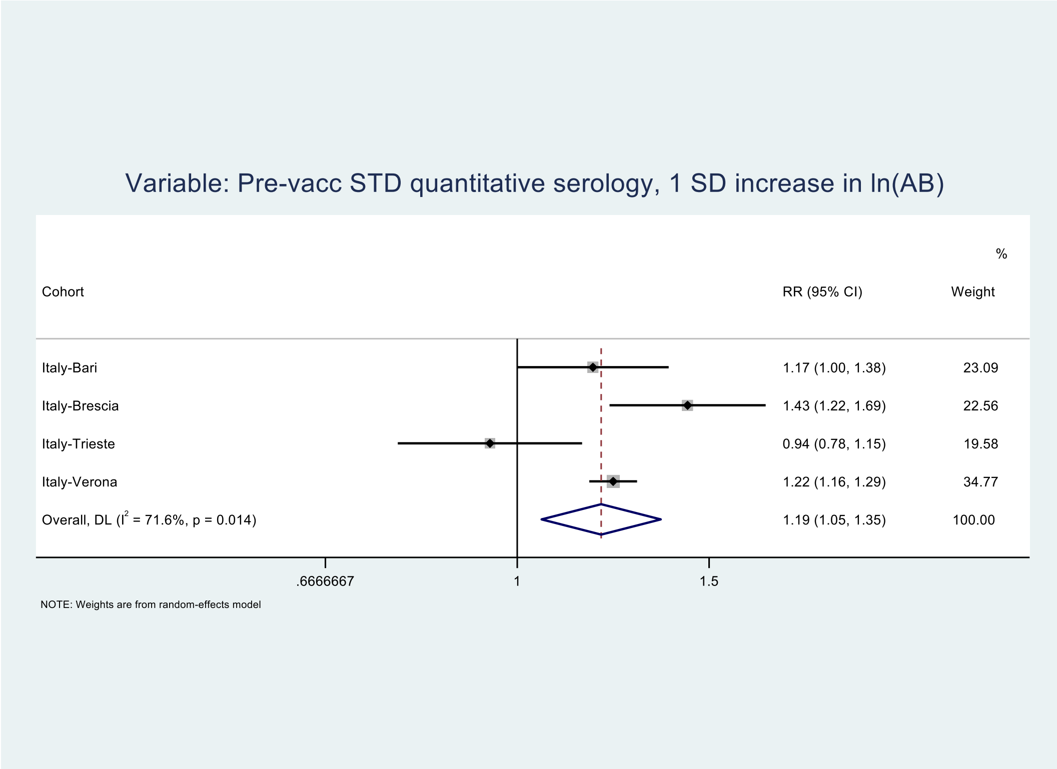

Relative risks and 95% confidence intervals of one SD increased in normalized antibody level
